# Taxonomic variations in the gut microbiome of gout patients with and without tophi might have a functional impact on urate metabolism

**DOI:** 10.1186/s10020-021-00311-5

**Published:** 2021-05-24

**Authors:** Eder Orlando Méndez-Salazar, Janitzia Vázquez-Mellado, Carlos S. Casimiro-Soriguer, Joaquin Dopazo, Cankut Çubuk, Yessica Zamudio-Cuevas, Adriana Francisco-Balderas, Karina Martínez-Flores, Javier Fernández-Torres, Carlos Lozada-Pérez, Carlos Pineda, Austreberto Sánchez-González, Luis H. Silveira, Ana I. Burguete-García, Citlalli Orbe-Orihuela, Alfredo Lagunas-Martínez, Alonso Vazquez-Gomez, Alberto López-Reyes, Berenice Palacios-González, Gabriela Angélica Martínez-Nava

**Affiliations:** 1 Unidad de Vinculación Científica de la Facultad de Medicina UNAM-INMEGEN, Instituto Nacional de Medicina Genómica, Periferico Sur 4809, Arenal Tepepan, Tlalpan, 14610 Mexico City, Mexico; 2 Programa de Doctorado en ICES, Facultad de Química, UNAM, Mexico City, Mexico; 3 Rheumatology Department, Hospital General de México Eduardo Liceaga Mexico City, Mexico City, Mexico; 4 Clinical Bioinformatics Area, Fundación Progreso y Salud (FPS). CDCA, Hospital Virgen del Rocio, 41013 Sevilla, Spain; 5 Computational Systems Medicine, Institute of Biomedicine of Seville (IBIS), Hospital Virgen del Rocio, 41013 Sevilla, Spain; 6 Bioinformatics in Rare Diseases (BiER), Centro de Investigación Biomédica en Red de Enfermedades Raras (CIBERER), FPS, Hospital Virgen del Rocío, 41013 Sevilla, Spain; 7 FPS/ELIXIR-Es, Hospital Virgen del Rocío, 42013 Sevilla, Spain; 8 Clinical Pharmacology, William Harvey Research Institute, Queen Mary University of London, London, EC1M 6BQ UK; 9 Laboratorio de Líquido Sinovial, Instituto Nacional de Rehabilitación “Luis Guillermo Ibarra Ibarra”, Calz México-Xochimilco 289, Arenal de Guadalupe, 14389 Mexico City, Mexico; 10 Servicio de Reumatología, Instituto Nacional de Rehabilitación “Luis Guillermo Ibarra Ibarra”, Mexico City, Mexico; 11 División de Enfermedades Musculo-Esqueléticas y Reumáticas, Instituto Nacional de Rehabilitación “Luis Guillermo Ibarra Ibarra”, Mexico City, Mexico; 12 Secretaria de Salud del Estado de Tlaxcala, Mexico City, México; 13 Departamento de Reumatología, Instituto Nacional de Cardiología Ignacio Chávez, Mexico City, Mexico; 14 Departamento de Epidemiología Genética, Centro de Investigaciones Sobre Enfermedades Infecciosas, Instituto Nacional de Salud Pública, Morelos, Mexico; 15 Hospital General Regional No. 1 “Ignacio García Tellez”, Instituto Mexicano del Seguro Social, Mérida, Yucatán Mexico; 16 Laboratorio de Gerociencias, Instituto Nacional de Rehabilitación “Luis Guillermo Ibarra Ibarra”, Mexico City, Mexico

**Keywords:** Gout, Gut microbiota, Uric acid metabolism

## Abstract

**Objective:**

To evaluate the taxonomic composition of the gut microbiome in gout patients with and without tophi formation, and predict bacterial functions that might have an impact on urate metabolism.

**Methods:**

Hypervariable V3–V4 regions of the bacterial 16S rRNA gene from fecal samples of gout patients with and without tophi (n = 33 and n = 25, respectively) were sequenced and compared to fecal samples from 53 healthy controls. We explored predictive functional profiles using bioinformatics in order to identify differences in taxonomy and metabolic pathways.

**Results:**

We identified a microbiome characterized by the lowest richness and a higher abundance of *Phascolarctobacterium*, *Bacteroides*, *Akkermansia*, and *Ruminococcus_gnavus_group* genera in patients with gout without tophi when compared to controls. The *Proteobacteria* phylum and the *Escherichia-Shigella* genus were more abundant in patients with tophaceous gout than in controls. Fold change analysis detected nine genera enriched in healthy controls compared to gout groups (*Bifidobacterium, Butyricicoccus, Oscillobacter, Ruminococcaceae_UCG_010, Lachnospiraceae_ND2007_group, Haemophilus, Ruminococcus_1, Clostridium_sensu_stricto_1, and Ruminococcaceae_UGC_013*). We found that the core microbiota of both gout groups shared *Bacteroides caccae*, *Bacteroides stercoris ATCC 43183*, and *Bacteroides coprocola DSM 17136*. These bacteria might perform functions linked to one-carbon metabolism, nucleotide binding, amino acid biosynthesis, and purine biosynthesis. Finally, we observed differences in key bacterial enzymes involved in urate synthesis, degradation, and elimination.

**Conclusion:**

Our findings revealed that taxonomic variations in the gut microbiome of gout patients with and without tophi might have a functional impact on urate metabolism.

**Supplementary Information:**

The online version contains supplementary material available at 10.1186/s10020-021-00311-5.

## Introduction

Gout, the most common form of inflammatory arthritis is the result of chronic hyperuricemia and the subsequent monosodium urate (MSU) crystal formation and deposition in articular cartilage and other joint and extra-articular tissues, which triggers an inflammatory response (Dalbeth et al. 2019; Narang et al. 2019). The clinical presentation includes frequent acute flares and chronic MSU crystal deposition, named tophus (Dalbeth et al. 2007). Chronic hyperuricemia is the result of environmental factors and a genetic background for urate production, as well as renal and intestinal excretion (Hn et al. 2019; Bobulescu and Moe 2012; Xu et al. 2016). Although evolutionary and adaptive processes in humans have restrained uricase production, responsible for urate degradation into allantoin (Alvarez-Lario and Macarron-Vicente 2010; Ramazzina et al. 2006), certain bacteria residing in the gut can metabolize one-third of the daily urate load produced endogenously and the exogenous urate from dietary purines (Sorensen and Levinson 1975; Maiuolo et al. 2016). Shao et al. found that the signatures of fecal microbiome and metabolome in gout patients can be characterized by disorders of metabolites involved in urate excretion and shifts in amino acids directly responsible of purine nucleoside biosynthesis. They also identified an enrichment of opportunistic pathogens and a decreased α diversity (Shao et al. 2017). Another study revealed gut dysbiosis in gout patients and demonstrated that *Bacteroides* were common and abundant in their gut microbiomes compared to healthy individuals (Guo et al. 2015). Lim et al. showed that *Bacteroides* possessed an enrichment of the enzyme 5-hydroxysourate hydrolase, which plays a crucial role in gut uricolysis (Lim et al. 2014). To date, no study has addressed the characterization and differentiation of the gut microbiome of gout patients with at least one subcutaneous tophus (detectable by physical examination) (Bursill et al. 2019, and without tophi, let alone in a western population. In this study, we present a robust description of the gut bacterial microbiome of Mexican gout patients and compare it in two different disease states of gout, in order to test the hypothesis that patients with gout and with tophaceous gout have changes in the structure and functional profile linked to uric acid synthesis and degradation pathways of their intestinal microbiota compared to healthy subjects.

## Materials and methods

### Study subjects and patients involvement

We included 58 patients with a diagnosis of gout (2015 ACR/EULAR) (Neogi et al. 2015): 33 had gout and at least one subcutaneous tophi), 25 had gout without subcutaneous tophi. Additionally, 53 healthy controls were recruited. Gout patients were consecutive patients attending their regular visit at one of two hospitals (Hospital General de México “Eduardo Liceaga” (HGM) and Instituto Nacional de Rehabilitación “Luis Guillermo Ibarra Ibarra” (INRLGII)). Healthy controls were blood donors from the INRLGII blood bank. All subjects agreed to participate and signed an informed consent. Trained research staff interviewed and clinically examined all participants. Individuals with diagnosis of diabetes, chronic renal failure, other rheumatic disease including other crystalline arthropathy (different than MSU crystals), Cushing syndrome, and chronic gastrointestinal diseases were excluded from this study. Additionally, patients receiving antibiotics, or *antiparasitic* therapy, or who had diarrhea in the last three months were not included in the study. Blood and stool samples were obtained following the protocol and instructions for sample collection and transport. Participants answered a previously validated semi-quantitative food frequency questionnaire about diet and medications (data will be published in a separate article). This study followed all statements of the Helsinki Declaration and was approved by the Ethics and Research Committee of the INRLGII (INR28/15) and HGM (DI/18/404-A/03/004).

### Anthropometric and biochemical assessment

A blood sample was taken by venipuncture from all participants within a fasting period of 8–12 h. Glucose, cholesterol, triglycerides, and urate levels were measured by spectrophotometry in a microplate absorbance reader (iMArk, Bio-Rad, CA, EUA) and following the manufacturer’s instructions (DiaSys, Holzheim Germany). The body mass index (BMI) was calculated using standard anthropometric parameters.

### Sample collection, DNA extraction and sequencing

All participants were ask to provide a fresh stool sample (the first bowel movement of the day). Previously to the collection of the sample participants were instructed by trained personnel for the correct way of collecting the sample and provided with the material needed. All samples were transport at 4 °C and stored within the first 2 h at − 80 °C. DNA was extracted from each sample using the QIAamp DNA stool kit (QIAGEN, Hilden, Germany) according to the manufacturer’s protocol. Sequencing libraries were built following a two-step polymerase chain reaction (PCR) protocol suggested by Illumina (Illumina, San Diego, CA, USA). The presence of amplicons (~ 560-bp) was confirmed by gel electrophoresis on a 1.5% agarose gel. Each sample was then purified with QIAquick PCR purification kit (QIAGEN, Hilden, Germany) and quantified by using Agilent 4200 Tape Station System (Agilent, Santa Clara, CA, United States). The final libraries were pooled at 4 pM with the addition of 10% PhiX into the final pool and subsequently sequenced using the Illumina Miseq platform (Illumina, San Diego, CA, USA), following the manufacturer’s specifications.

### Bioinformatics analysis

All raw sequence reads were filtered under a strict quality preprocessing protocol to remove sequences with Phred quality score < Q30 using fastp tool v0.20.0 (Chen et al. 2016). After filtering, 91% of the sequencing reads reached Q30 (99.9% base call accuracy) for controls and cases sequences. Consequently, paired reads were merged using PEAR v0.9.10 (Zhang et al. 2014) with a threshold of 20 bp minimum overlap and imported into QIIME2, version 2019.4 (Bolyen et al. 2019). Afterwards, chimeras were removed, and sequences were denoised with *-p*-*trim-length* script, truncating reads at a position 250 using Deblur algorithm (Amir et al. 2017). Overall, we obtained 3,444,321 clean and filtered sequences with a mean count of 31,599 reads per sample.

Taxonomy assignment was performed against the SILVA v132 ribosomal reference database (Quast et al. 2012), clustered at 99% sequence similarity. The resulting amplicon sequence variants (ASVs) abundance table, metadata, and taxonomy data were imported into phyloseq (McMurdie and Holmes 2013) and analyzed with R Studio version 3.5.2 (http://www.rstudio.com).

The ASV table was rarefied to adjust the sampling depth among samples to the one with the lowest reads. Alpha diversity metrics for richness (observed species, Chao1, ACE) and diversity (Shannon and Simpson) were estimated and compared using phyloseq. For multiple comparison adjustment we calculated the false discovery rate (FDR) using the Hommel method. In addition, we analyzed richness and diversity indices removing BMI ≥ 30 kg/m^2^ (five samples from the gout group and three from the tophaceous gout group).

Relative log expression (RLE) normalization method to test for differential abundance of ASVs was performed in DESeq2 (Love et al. 2014). To visualize the variations in bacterial community composition according to studied groups’ metadata, we calculated beta-diversity with a principal coordinate analysis (PCoA) of Bray–Curtis dissimilarity based on ASV composition. We used a permutational multivariate analysis of variance (PERMANOVA) to test differences between groups, and p < 0.05 was taken as statistical significance. For the taxonomic composition analysis and visualization, stats and ggplot2 R packages were used. The Kruskall-Wallis test was first applied to compare ASVs among groups; only if the Kruskall-Wallis test showed a p < 0.05 was the Wilcoxon sum-rank test performed to compare pairs of groups. We generated boxplots of the ASV relative abundances using amp_boxplot function (within the ampvis2 package v.2.5.9) (Andersen et al. 2018). Fold changes were also calculated by dividing the median values of group abundances for significant results obtained from the Wilcoxon analysis. Besides, a Linear Discriminant Analysis (LDA) was performed with MASS package in R to test whether the taxa abundances detected in the non-parametric tests and fold changes were able to discriminate between groups. Based on an abundance ratio parameter of 0.8, and an ASVs removal with an abundance < 0.01% of the total read count proposed by Astudillo-García (Astudillo-García et al. 2017), we generated a Venn diagram and Krona plots to show unique and common ASVs among groups.

We used an in silico approach to infer bacterial protein–protein interaction (PPI) networks, using the STRING-DB version 11.0 (https://string-db.org), and to predict the role that bacterial species shared between gout groups might play in purine metabolism. Statistical significance was considered with p-values under 0.05. The resulting ASV table was used to predict KEGG Ortholog (KO) functional profiles of bacterial communities from 16S rRNA data using the Tax4Fun2 R package (https://sourceforge.net/projects/tax4fun2/).

We applied a two-sided Welch’s t-test in STAMP (Statistical Analysis of Metagenomic Profiles) (Parks and Beiko 2013) to establish statistical differences and to determine possible enriched bacterial functions among groups.

## Results

### Differentially abundant taxa and alpha diversity among study groups

Additional file 1: Table S1 illustrates the anthropometric and biochemical features of patients and controls; BMI, glucose, and triglycerides were higher in gout patients and tophaceous gout patients than in healthy controls.

**Fig. 1:**
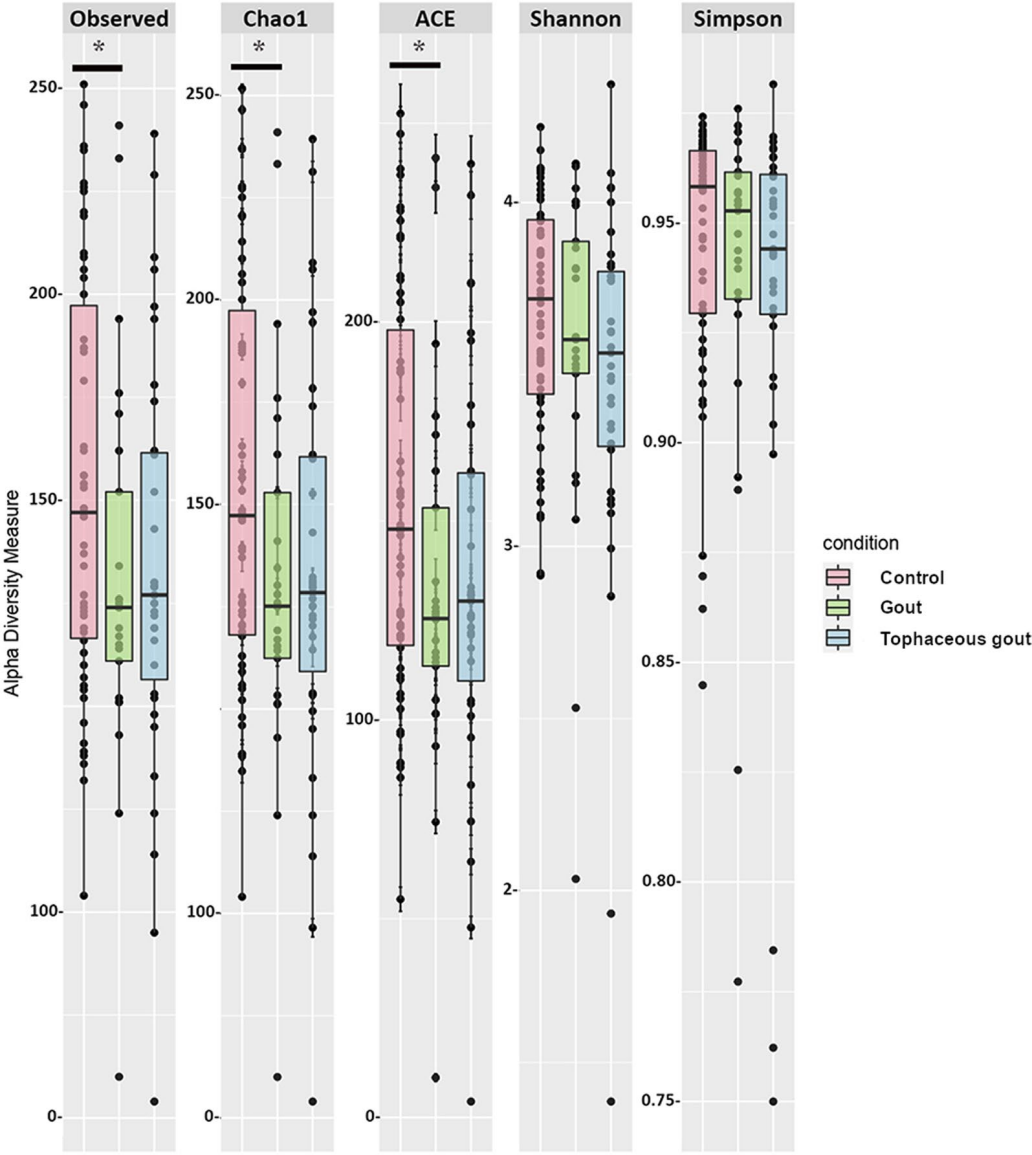
Alpha-diversity plots of bacterial communities in the microbiome of study groups. Variations for richness (Observed, Chao1, ACE) and diversity (Shannon and Simpson) indexes between both gout groups and healthy controls. *Significant difference after multiple comparison adjustment

**Fig. 2:**
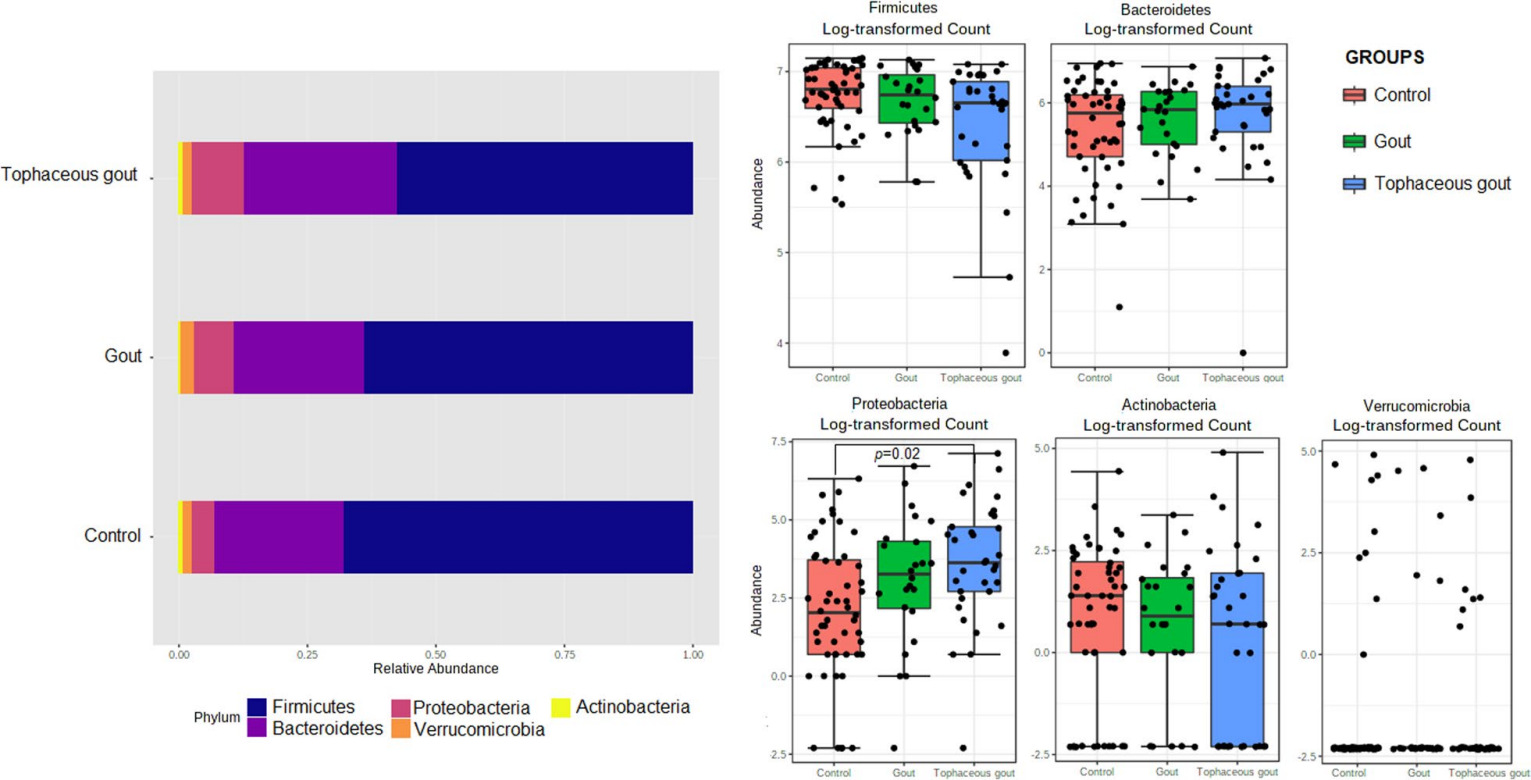
Relative abundance of ASVs at the phylum level. Stacked bar plots represent the relative abundance at the phylum level using SILVA 132 database. Boxplots showing comparative distribution of *Firmicutes, Bacteroidetes, Proteobacteria, Actinobacteria and Verrucomicrobia* in controls (red bars), in gout group (green bars) and in the tophaceous gout group (blue bars). Boxes denote the interquartile range (IQR) between the first and third quartiles, and the line inside represents the median (2nd quartile). Whiskers show the lowest and the highest values

**Fig. 3:**
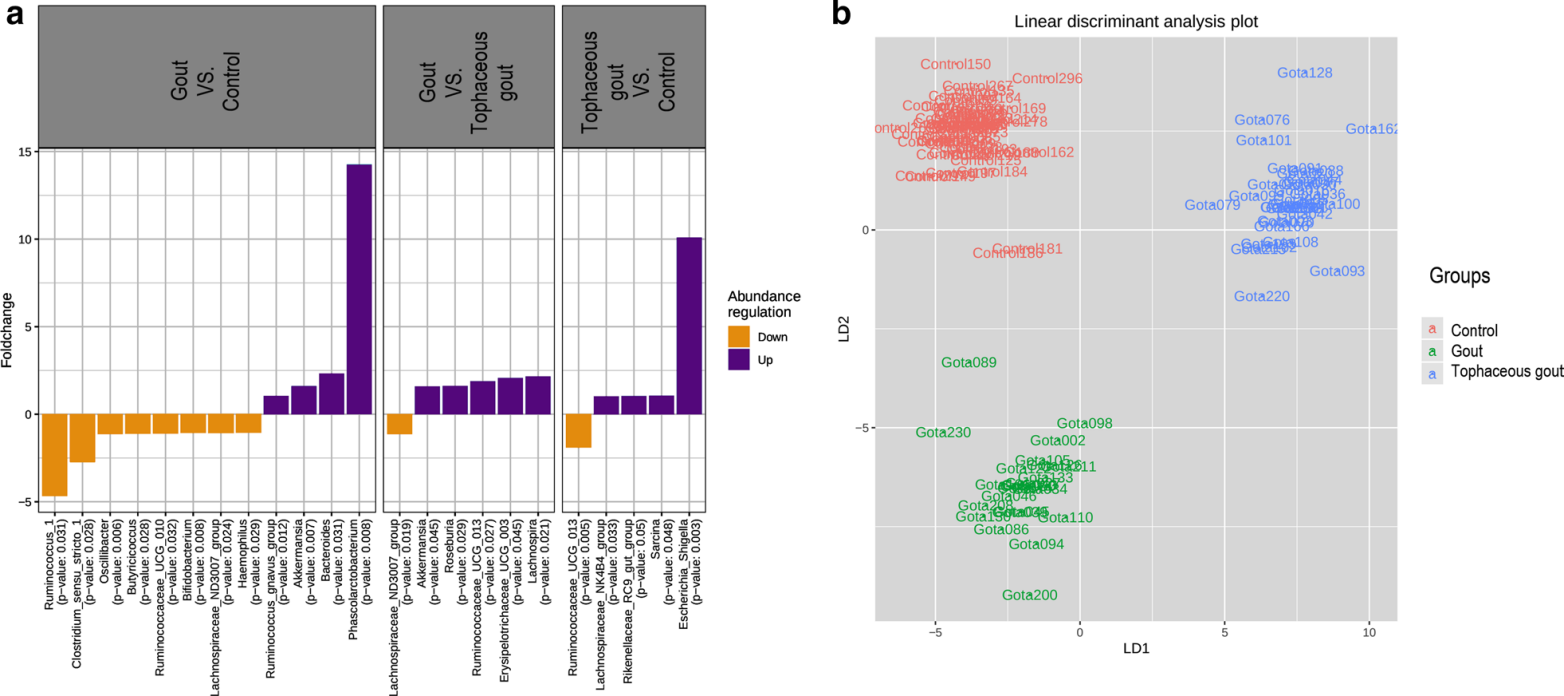
**a** Fold change of the relative abundances of bacterial genera showing significant differences between both gout groups and healthy controls. Purple color (positive fold change) and orange color (negative fold change) indicate an increase or a decrease of the bacterial genera within each group, respectively. **b** Linear discriminant analysis (LDA) plot of genera abundances data derived from non-parametrical statistical analysis shows the significance of genera combinations in differentiating the three study groups

The following richness indices were significantly increased in healthy controls compared to gout patients: Chao1 (p = 0.049), Observed species (p = 0.041), and ACE (p = 0.044) (Fig. 1). After multiple comparison adjustment, the difference seen in Chao1, Observed species and ACE between control group and gout patients remained significant (p = 0.049). Rarefaction curves for each sample are shown in (Additional file 2: Fig. S1). Nonetheless, no statistical difference was found in the Shannon and Simpson diversity indices. Tophaceous gout patients exhibited no statistical variation in any of the α-diversity parameters. Alpha diversity analysis without patients with BMI ≥ 30 kg*/*m^2^ provided substantial confirmation of the findings obtained in Fig. 1 (Additional file 3: Fig. S2 A). Moreover, beta-diversity was non-significantly different between the study groups (Additional file 3: Fig. S2B). Sequences were assigned to 5349 amplicon sequence variants (ASVs) at ≥ 99% similarity and clustered into 5 phyla, 9 classes, 11 orders, 21 families, and 63 genera. At the phylum level, *Proteobacteria* was significantly more abundant in tophaceous gout patients than in healthy controls (p < 0.05) (Fig. 2). The relative abundance of the 50 most abundant genera is shown in Additional file 4: Fig. S3. Pairwise comparison based on a fold change cut-off ≥  ± 1.5 revealed twenty differentially abundant taxa at the genus level among groups. Of these, nine genera were more abundant in the control group, eight were overrepresented in gout group, and five were enriched in the tophaceous group (Fig. 3a). Lineal Discriminant Analysis showed that *Ruminococcus_1*, *Clostridium_sensu_stricto_1, Oscillibacter, Butyricicoccus, Ruminococcaceae_UCG_010, Bifidobacterium, Lachnospiraceae_ND3007_group, Haemophilus, and Ruminococcaceae_UGC_013* were the detected genera in the non-parametric tests and fold changes that can discriminate between the control group from the two gout groups. Moreover, *Phascolarctobacterium, Bacteroides, Lachnospira, Erysipelotrichaceae_UCG_003, Ruminococcaceae_UGC_013, Roseburia, Akkermansia, and Ruminococcus_gnavus_group,* have the ability to discriminate between gout group, from controls and tophaceous gout group. While, *Escherichia-Shigella, Sarcina, Rikenellaceae_RC9, Lachnospiraceae_NK4B4, and Lachnospiraceae_ND3007* were the combination of genera that best discriminate tophaceous gout from the other groups (Fig. 3b).

### Defining a core microbiota of gout

**Fig. 4:**
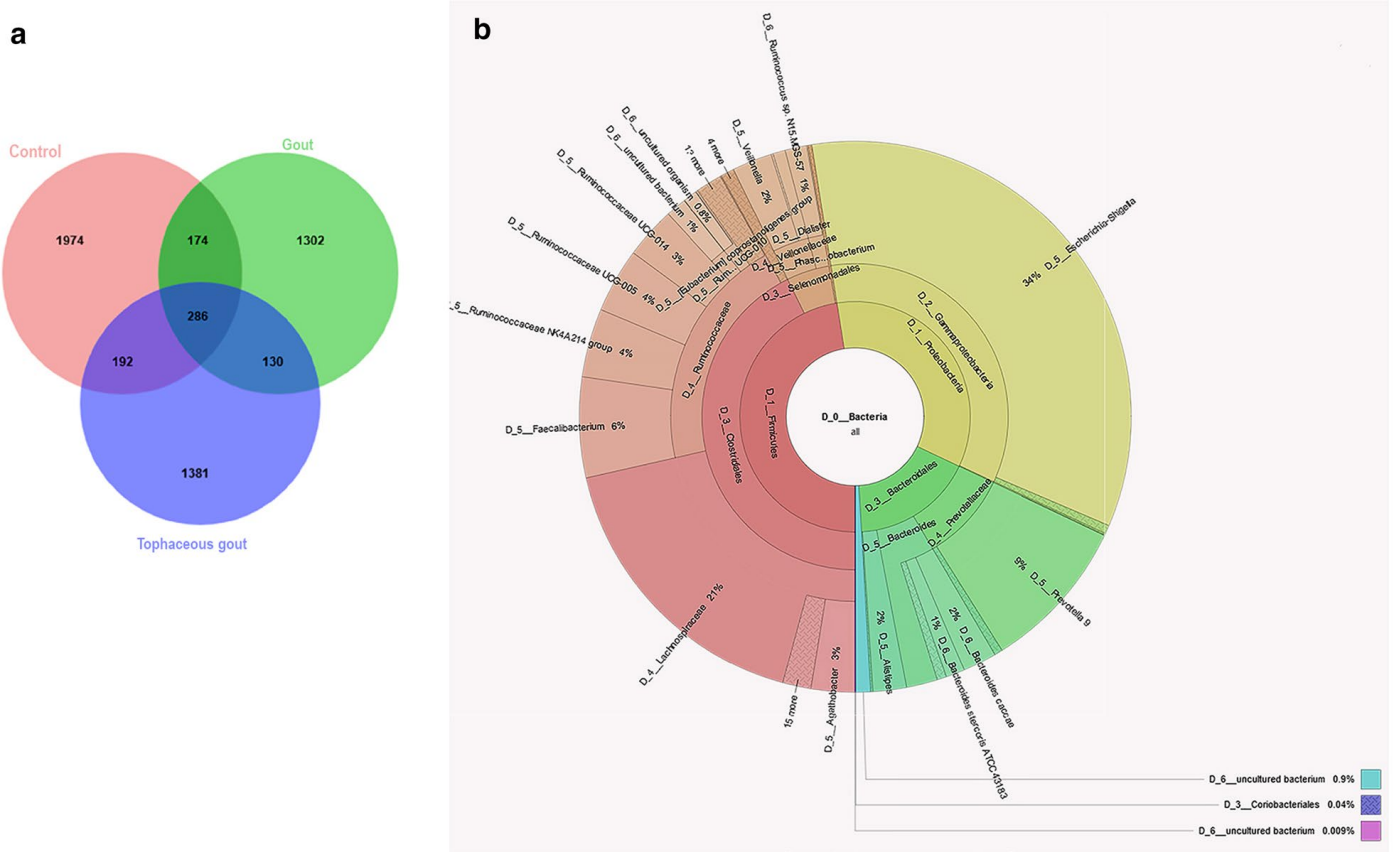
**a** Venn diagram showing the number of shared and unique core ASVs among the three study groups. The core microbiota in the gout groups is exhibited in the overlap of the green and blue circles. **b** Krona chart representing the taxonomic composition and relative abundance of the most abundant ASVs found in the gout groups

Based on a 0.01 relative abundance cut-off, we observed that both gout groups (gout patients and tophaceous gout patients) shared 130 overlapping ASVs (Fig. 4a). Of the total ASVs detected, 1302 represented an exclusive composition in gout patients, whereas 1381 ASVs belonged to tophaceous gout patients, and 1974 were unique to healthy controls; interestingly, 286 ASVs were common to all samples. Krona charts were used to represent the relative taxonomic contribution (ASV percentages) from all samples (Additional file 5: Fig. S4).

The most abundantly shared ASVs between both gout groups at the genus level were *Faecalibacterium*, *Ruminococcaceae_NK4A214_group*, *Ruminococcaceae_UCG-005*, *Ruminococcaceae_UCG-14*, *Veillonella*, *Phascolarctobacterium*. Interestingly, *Prevotella_9* and *Bacteroides* (both from the *Bacteroidales* family) were the most representative genera of this phylum among these two groups. The most representative *Proteobacteria* genus was *Escherichia-Shigella* (34%) (Fig. 4b). We found a core of three most abundant ASVs within the genus *Bacteroides* that included *Bacteroides caccae*, *Bacteroides stercoris ATCC 43,183*, and *Bacteroides coprocola DSM 17,136* (Additional file 5: Fig. S4A). We identified 174 and 192 ASVs shared among healthy controls/gout patients (Additional file 5: Fig. S4B) and healthy controls/tophaceous gout patients, respectively (Additional file 5: Fig. S4C).

### Functional prediction of bacteria in the core microbiota of gout patients is linked to the synthesis of intermediate metabolites for purine formation

**Fig. 5:**
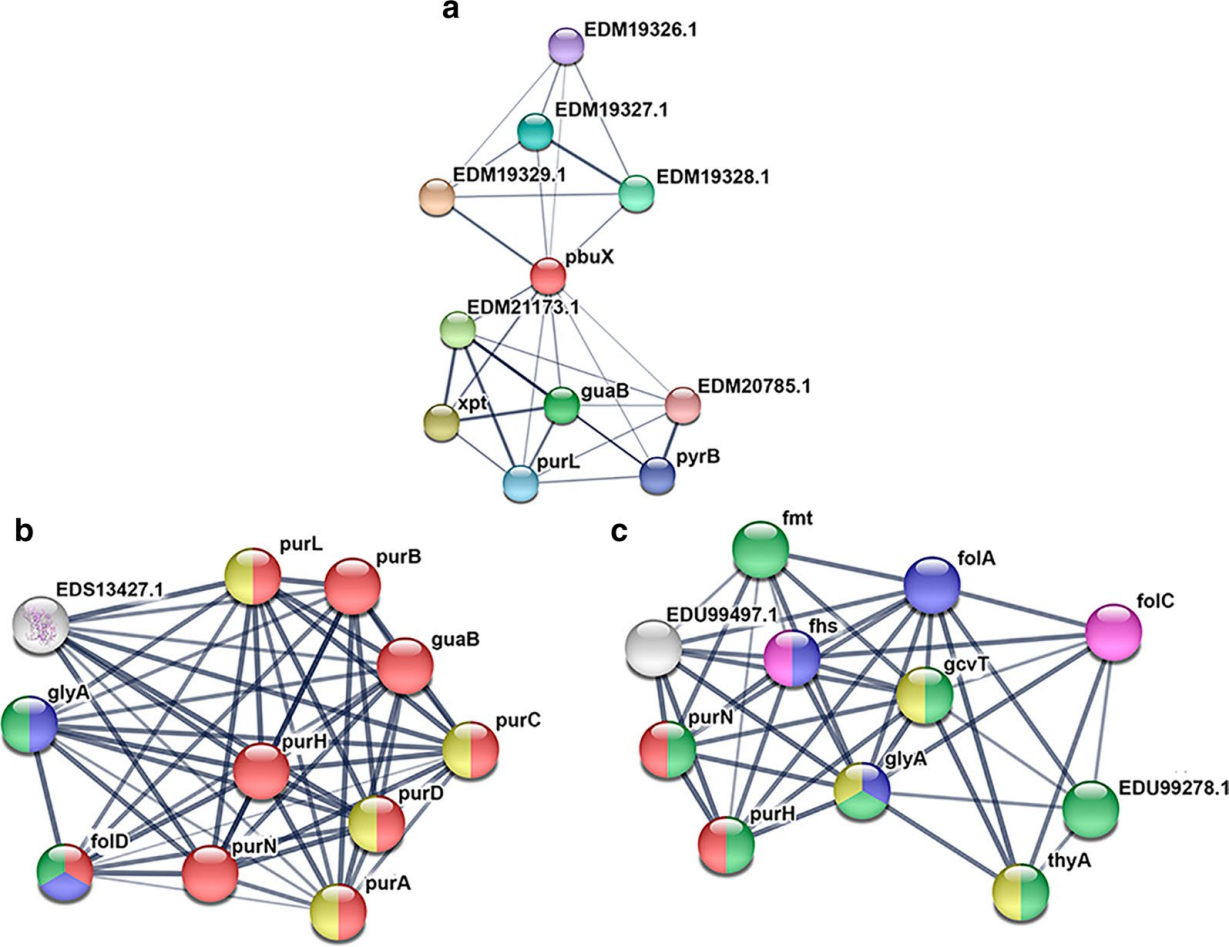
Protein–protein interaction analysis predicted by STRING. Nodes are colored based on their specific role: purine biosynthesis (red), one-carbon metabolism (blue), nucleotide-binding (yellow), ligases (pink), and amino acid biosynthesis (green). Proteins involved in multiple functions are filled with various colors. Thicker lines define the most robust associations. **a** *Bacteroides caccae* showed significant enrichment of proteins strongly involved in purine metabolism. **b** *Bacteroides stercoris ATCC 43183*, displayed functional categories included purine biosynthesis, one-carbon metabolism, nucleotide-binding, and amino-acid biosynthesis. **c** For *Bacteroides coproccola DSM 17136* and *Bacteroides stercoris ATCC 43183* we identified protein–protein interaction in enzymes involved in one-carbon metabolism, purine biosynthesis, as well as methyltransferases, transferases, and ligases

We explored a functional protein association network focusing on three species of bacteria (*Bacteroides caccae*, *Bacteroides stercoris ATCC 43183*, and *Bacteroides coproccola DSM 17136*) shared between both gout groups (Fig. 5a–c). The STRING analysis *of Bacteroides caccae* revealed a significant enrichment (p = 0.0001) of protein–protein interaction (PPI) among eleven proteins. Five of these functional pairs were proteins involved in purine metabolism (pbux (xanthine permease), guaB (inosine-5′ monophosphate dehydrogenase), purL (phosphoribosylformylglycinamidine synthase), xpt (xanthine phosphoribosyltransferase)). This enrichment indicates that proteins are partially biologically connected and actively involved in purine metabolism (FDR = 0.0003), GMP biosynthesis (FDR = 0.0009), and glutaminase activity (glutamine amidotransferase) (FDR = 0.004) (Fig. 5a).

For *Bacteroides stercoris ATCC 43183*, the proteins detected were significantly enriched in four pathways: purine biosynthesis, one-carbon metabolism, nucleotide-binding, and amino-acid biosynthesis, with a FDR of 2.55e−18, 0.002, 0.016, and 0.043, respectively. The proteins detected were: bifunctional purine biosynthesis protein (PurH), AICAR transformylase/IMP cyclohydrolase (PurH), serine hydroxymethyltransferase (glyA), adenylosuccinate synthetase (purA), phosphoribosylamine-glycine ligase (purD), phosphoribosylglycinamide formyltransferase (purN), 5-phospho-ribosyl-N-formylglycinamide (purL), phosphoribosylaminoimidazole-succinocarboxamide (SAICAR) synthase (purC), and inosine-5′-monophosphate dehydrogenase (guaB) (Fig. 5b).

A shared PPI network for three targeted proteins that are also involved in purine biosynthesis (FDR = 0.002), and three proteins that participates in one carbon metabolism (FDR = 1.76e−05) was detected between *Bacteroides coprocola DSM 17136* and *Bacteroides stercoris ATCC 43183*. Moreover, *B. coprocola DSM 17136* had an enrichment in protein–protein interaction that included three methyltransferases (glyA, gcvT, and thyA) (FDR = 0.001), six transferases (purN, purH, glytA, gcvT, EDU99278.1, and fmt) (FDR = 4.60e−05) and two ligases (fhs and folC) (FDR = 0.030) (Fig. 5c).

### Variations in key bacterial enzymes among study groups

**Fig. 6:**
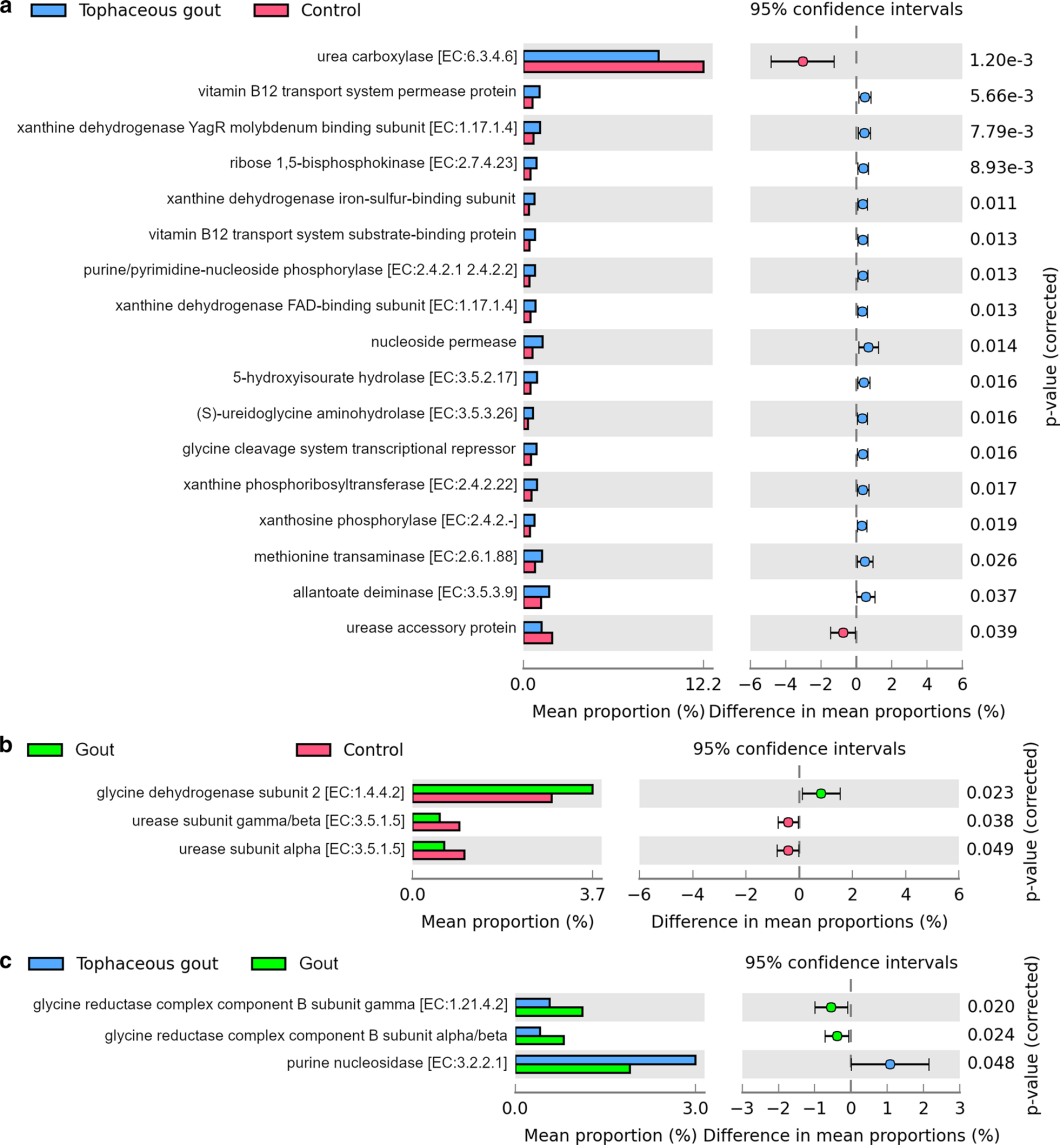
Stacked column bar graph representing the predicted metabolic characteristics that differentiate between **a** tophaceous gout patients vs. healthy controls, **b** gout patients vs. healthy controls, and **c** tophaceous gout patients vs. gout patients. Differences were considered significant at p-value < 0.05 using Welch’s t-test

We observed 47 KEGG functional orthologues related to the oxidative metabolism of purines, metabolic pathways for purine biosynthesis, vitamin B12 transport, and urate excretion that were significantly different between groups (Fig. 6a–c).

Vitamin B12 (permease protein and substrate binding protein), nucleoside permease, glycine cleavage system transcriptional repressor, several xanthine dehydrogenase iron-sulfur-binding subunits, xanthine and nucleoside phosphorylases, methionine transaminase, oxidoreductases, transferases, hydrolases were much higher in the fecal microbiome of tophaceous gout patients compared to healthy controls. Urea carboxylase and urease accessory protein were more enriched in healthy controls (Fig. 6a).

The KEGG pathway analysis also detected significant differences in microbiome functions between healthy controls and the gout patients. Glycine dehydrogenase subunit was the only enzyme enriched in gout patients compared to healthy controls. Two urease subunits were more abundant in the microbiome of healthy controls compared to gout patients (Fig. 6b).

The abundance of glycine reductase complex component B subunits alpha, beta and gamma was significantly higher in gout patients than tophaceous gout patients, whereas purine nucleosidase abundance was higher in tophaceous gout patients (Fig. 6c).

KEGG enzyme and orthology identifier for bacterial proteins that were significantly different among study groups are in Additional file 6: Table S2.

## Discussion

Despite accounting for only one-third of total urate excretion, the impact of intestinal urate excretion in gout disease is scarcely understood. Recent investigations have shown a recurrent pattern associated with alterations in bacterial species richness, and in their crucial functions in the intestine. Our study found taxonomic variations and shifts among gout patients, tophaceous gout patients, and healthy controls regarding bacterial functions involved in urate metabolism.

Some studies suggest that low bacterial richness in the gut microbiome is associated with chronic diseases and a more pronounced inflammatory phenotype (Chatelier et al. 2013). Notably, our analysis showed a lower species richness in gout patients than in healthy controls. Our results are consistent with previous reports that found lower bacterial diversity in Chinese gout patients, suggesting intestinal dysbiosis (Shao et al. 2017).

We found a significantly higher abundance of butyrate-producing bacteria (*Butyricicoccus* and *Oscillibacter*) in healthy controls. This is the first time that the genus *Butyricicoccus* is reported as significantly overrepresented in healthy controls compared to gout patients. Healthy controls also exhibited an enrichment of previously reported beneficial genera, such as *Bifidobacterium*, *Clostridium_sensu_stricto_1*, and *Ruminococcus_1*. Interestingly, the coexistence of butyrate-producing and *Bifidobacterium* genera may contribute to the maintenance of the intestinal barrier, as well as the immunomodulatory and anti-inflammatory processes (Riviere et al. 2016; Devriese et al. 2017; Krumbeck et al. 2018). Zhuang et al*.* reported similar findings of metagenomic species enriched in the gut microbiome of healthy controls (*Clostridium* butyrate-producing bacteria and *Bifidobacterium pseudocatenulatum*). They also observed that *Ruminococcus* abundance was negatively associated with gout disease (Guo et al. 2015).

*Phascolarctobacterium* and *Bacteroides* were the most enriched genera in both gout groups. These genera have been found more abundantly in the intestinal tract of a hyperuricemic rat model (Liu et al. 2020). *Bacteroides* is a gut enterotype reported to promote urate conversion into allantoin, and hence might be involved in serum urate level regulation in humans (Lim et al. 2014). The *Akkermansia* genus, which we also found more abundantly in Gout patients than in healthy controls and tophaceous gout patients, was previously reported in higher abundance among young patients with enthesitis-related arthritis (Stoll et al. 2019).

Additionally, we identified a “core microbiota” for the two gout groups encompassing three *Bacteroides* species. A recent study reported enrichment of *Bacteroides* and in particular *Bacteroides caccae* in gout patients (Shao et al. 2017).

Yamauchi et al. demonstrated how some bacterial species within the gut microbiome could release many metabolites and protein molecules that distinctively impact host purine levels (Yamauchi et al. 2020). Given that enrichment of *Bacteroides* may be the footprint of gout disease, our work implied an understanding of the role of *Bacteroides* species found in the core microbiota of gouty patients. We identified multiple proteins that orchestrate de novo synthesis, salvage, and formation of purine intermediates with protein networks stored in STRING.

The fecal microbiome of male gout patients was also found to be enriched in certain amino acids, including glycine. Reductive glycine pathway could be a critical metabolic route for assimilating one-carbon compounds (Yishai et al. 2018). One-carbon metabolism mediated by folate cofactor supports purine biosynthesis and amino acid homeostasis, including glycine, serine, and methionine (Ducker and Rabinowitz 2017). Consistent with the reports on Chinese patients (Shao et al. 2017), our findings show that both glycine dehydrogenase and glycine reductase were significantly higher in gout patients compared to healthy controls and tophaceous gout patients. Moreover, enzymes involved in methionine transamination, vitamin B12 transport system, and glycine cleavage system were increased in tophaceous gout patients compared to healthy controls. Therefore, we believe that the gut microbiome of gout patients not only participates in nucleotide salvage and de novo purine biosynthesis pathways, but also performs essential functions in the de novo synthesis of purine precursors (such as glycine), as well as in the synthesis of 10f-formyltetrahydrofolate.

The purine nucleosidase enzyme that we observed to be differentially abundant in tophaceous gout patients compared to gout patients is involved in the formation of nucleobases (Kopecna et al. 2013). In 2006, Ogawa reported that a high nucleoside activity of some anaerobic bacteria residing in the gut of hyperuricemic rats helped lower the elevated serum urate levels in these animals. Therefore, purine nucleoside may play a specific role in serum urate level modulation in tophaceous gout patients (Ogawa 2006).

When compared to healthy controls, tophaceous gout patients had an enrichment of *Proteobacteria*, accompanied by significant increases in *Escherichia-Shigella*, *Sarcina*, *Rikenellaceae_RC9*, and *Lachnospiraceae_NK4B4*. Similar results were previously reported (Xi et al. 2019), where a high proliferation of *Proteobacteria* (particularly *Escherichia-Shigella*) was found in the gut microbiota of animals lacking urate oxidase. Thus, the presence of these bacteria might play a crucial role in the development of gout. Additionally, it is well known that enrichment of *Proteobacteria* may represent the dysbiosis signature in the gut microbiota (Shin et al. 2015). We have elucidated the enrichment of three bacterial xanthine dehydrogenase isoforms with oxidoreductase activity in tophaceous gout patients. A previous study showed an increase in xanthine dehydrogenase in gout patients (Guo et al. October 2015). This enzyme plays key roles in the successive oxidation of hypoxanthine to xanthine and xanthine to urate (Wang et al. 2016). Urate is oxidized, producing 5-hydroxysourate, allantoin, (S)-ureidoglycine, and the end product of purine catabolism, glyoxylate, in order to yield oxalurate and glycine (Ramazzina et al. 2010). This process enables gut bacteria to obtain carbon and nitrogen. Hualin Xi et al. revealed that bacterial lineages belonging to *Escherichia-Shigella*, which were enriched among tophaceous gout patients in our study, might contribute to purine salvage by inducing the xanthine dehydrogenase activity (Xi et al. 2000). They also proposed that this action may allow these bacteria to obtain nitrogen as a means of survival. Another important finding was the enrichment of the enzymes involved in the final purine catabolism (5-hydroxyisourate hydrolase, allantoate deiminase, and (S)-ureidoglycine aminohydrolase) in tophaceous gout patients versus healthy controls. There is evidence of an adaptive response of *Escherichia-Shigella* members to long-term nitrogen starvation in anaerobic environments, which requires allantoin degradation (Switzer et al. 2020). It is worth noting that *E. coli* can obtain four nitrogen atoms from purines, while it can only gain one nitrogen atom from each uracil molecule through the reductive pyrimidine catabolic pathway (Salway 2018; Yin et al. 2019). We hypothesized that the increased activity of xanthine dehydrogenase and the enzymes of ureide synthesis in tophaceous gout patients is likely an adaptive process of the gut microbiome, which may be attributable to more than 8 years of allopurinol treatment. This antihyperuricemic drug inhibits urate production and, hence, allantoin formation by bacteria (Yang et al. 2012).

Finally, ureases were the most elevated enzymes in healthy controls compared to gout patients, suggesting that intestinal microbiota ureases may play a crucial role in the final degradation of purine and ureides in order to produce urea. Urea hydrolysis enables nitrogen acquisition as a valuable energy source (Kanamori et al. 2004; Mora and Arioli 2014).

A remarkable limitation of our study is that 16s rRNA data do not allow direct inference of genes from the bacterial population found. Therefore, future research should be carried out with metagenomic shotgun sequencing and metatranscriptomics analysis. This would enable more accurate inferences about bacterial structure and real gene expression to identify the strain-specific genomic features. Another limitation that is worth mentioning is that BMI was significantly different among our study groups. In Mexico, gout tends to be more severe than in other countries, and obesity is frequently associated with this type of inflammatory arthritis, especially among subjects with a shorter duration of the disease (Vázquez-Mellado et al. 2006). The patients included in our study showed higher BMI than control group, especially the group of gout patients. We considered this an intrinsic limitation given the behavior of the disease. Nonetheless, we think this does not affect our results, given that the composition of the gut microbiota of gout patients was found to be enriched with bacterial genus reported in some studies as decreased in obese individuals (*Bacteroides* and *Akkermansia*) (Crovesy et al. 2020). Furthermore, we obtained similar findings in alpha diversity indices with rarefaction excluding samples from patients with a BMI ≥ 30 kg/m^2^, suggesting that the lower diversity observed in gout patients is not due to obesity but to gout itself.

On the other hand, despite the differences observed in serum glucose levels, we avoided the inclusion of those individuals with fasting glucose > 126 mg/dL since it would indicate diabetes diagnosis according to the American Diabetes Association (ADA) (previously listed in the exclusion criteria). This strategy helped us to avoid including patients with both gout and type 2 diabetes (T2D**)**. Nonetheless, gout is a highly complex metabolic disease, and the positive correlation between hyperuricemia and hyperglycemia has been long studied (Griffiths 1950). Despite that the etiological mechanisms behind this relationship are not fully understood, hyperuricemia has been proven to be the first metabolic disruption to appear in patients not only with T2D but also in metabolic syndrome (MetS) patients (Johnson 2015; Li et al. 2013), two of the most prevalent comorbidities of gout. This could be the reason for the significant difference we observed in glucose levels among controls and gout patients, and supports the notion that the results presented here reflects the truly metabolic disruption seen in gout patients.

## Conclusions

In conclusion, our results highlight the taxonomic composition differences of the gut microbiota between two gout groups at different stages of the disease compared to healthy subjects. In general, we found enrichment of distinct functions within the microbiome of both gout groups related to urate metabolism, one-carbon metabolism, and both amino acid and purine biosynthesis.

The gut microbiome has become an essential component for understanding gout complexity, and its shifts showed in this study may provide metabolic footprints of gout patients and healthy individuals. Further studies are needed to understand how to reestablish or inhibit gut microbiome functions in gout patients to achieve lower serum urate levels.

## Supplementary Information

**Additional file 1: Table S1.** Descriptive statistics and results of anthropometric, biochemical variables in gout groups and healthy controls. Text in bold denotes statistical significance. Age, BMI, and total cholesterol are expressed in mean and standard deviation, while the other continuous variables are summarized by median and interquartile range. a. P-value obtained from ANOVA. b. P-value obtained from Kruskall-Wallis test. c. P-value obtained from two tail Student’s T test. d. P-value obtained from Mann-Whitney’s U test. *Significant p-value obtained from Tukey post-hoc test comparing against control group & significant p-value obtained after multiple comparison correction comparing against control group.

**Additional file 2: Fig. S1.** Rarefaction analysis of the different samples.

**Additional file 3: Fig. S2.** A) Boxplot of Alpha-diversity indices without samples from patients with a BMI ≥ 30 kg/m2. S2B. B) Plot showed no clear clustering pattern between the gut microbiome of the study samples.

**Additional file 4: Fig. S3.** Boxplot showing the occurrence of the top 50 most abundant genera in Mexican patients with gout and healthy controls.

**Additional file 5: Fig. S4.** Krona charts representing the taxonomic composition of the most abundant ASVs shared between the study groups. A) ASVs at the genus and species level shared between the two gout groups. B) ASVs shared between healthy controls and gout patients. C) ASVs shared between healthy controls and tophaceous gout patients.

**Additional file 6: Table S2.**

## Data Availability

The data supporting the findings of this study are available on request from the corresponding author. The data are not publicly available due to privacy or ethical restrictions.

## References

[CR1] Alvarez-Lario B, Macarron-Vicente J (2010). Uric acid and evolution. Rheumatology.

[CR2] Amir A, McDonald D, Navas-Molina JA, Debelius J, Morton JT, Hyde E (2017). Correcting for microbial blooms in fecal samples during room-temperature shipping. mSystems.

[CR3] Andersen KS, Kirkegaard RH, Karst SM, Albertsen M. ampvis2: an R package to analyse and visualise 16S rRNA amplicon data. bioRxiv; 2018. https://madsalbertsen.github.io/ampvis2/index.html

[CR4] Astudillo-García C, Bell JJ, Webster NS, Glasl B, Jompa J, Montoya JM (2017). Evaluating the core microbiota in complex communities: a systematic investigation. Environ Microbiol.

[CR5] Bobulescu IA, Moe OW (2012). Renal transport of uric acid: evolving concepts and uncertainties. Adv Chronic Kidney Dis..

[CR6] Bolyen E, Rideout JR, Dillon MR, Bokulich NA, Abnet CC, Al-Ghalith GA (2019). Reproducible, interactive, scalable and extensible microbiome data science using QIIME 2. Nat Biotechnol..

[CR7] Bursill D, Taylor WJ, Terkeltaub R, Abhishek A, So AK, Vargas-Santos AB (2019). Gout, Hyperuricaemia and Crystal-Associated Disease Network (G-CAN) consensus statement regarding labels and definitions of disease states of gout. Ann Rheum Dis.

[CR8] Chen S, Zhou Y, Chen Y, Gu J (2018). fastp: an ultra-fast all-in-one FASTQ preprocessor. Bioinformatics.

[CR9] Crovesy L, Masterson D, Rosado EL (2020). Profile of the gut microbiota of adults with obesity: a systematic review. Eur J Clin Nutr.

[CR10] Dalbeth N, Collis J, Gregory K, Clark B, Robinson E, McQueen FM (2007). Tophaceous joint disease strongly predicts hand function in patients with gout. Rheumatology (oxford).

[CR11] Dalbeth N, Choi HK, Joosten LAB, Khanna PP, Matsuo H, Perez-Ruiz F (2019). Gout. Nat Rev Dis Prim..

[CR12] Devriese S, Eeckhaut V, Geirnaert A, Van den Bossche L, Hindryckx P, Van de Wiele T (2017). Reduced mucosa-associated butyricicoccus activity in patients with ulcerative colitis correlates with aberrant claudin-1 expression. J Crohns Colitis..

[CR13] Ducker GS, Rabinowitz JD (2017). One-carbon metabolism in health and disease. Cell Metab..

[CR14] Griffiths M (1950). The mechanism of the diabetogenic action of uric acid. J Biol Chrm.

[CR15] Guo Z, Zhang J, Wang Z, Ang KY, Huang S, Hou Q (2015). Intestinal microbiota distinguish gout patients from healthy humans. Sci Rep.

[CR16] Johnson RJ (2015). Why focus on uric acid?. Curr Med Res Opin..

[CR17] Kanamori T, Kanou N, Atomi H, Imanaka T (2004). Enzymatic characterization of a prokaryotic urea carboxylase. J Bacteriol..

[CR18] Kawamura Y, Nakaoka H, Nakayama A, Okada Y, Yamamoto K, Higashino T, Sakiyama M, Shimizu T, Ooyama H, Ooyama K, Nagase M (2019). Genome-wide association study revealed novel loci which aggravate asymptomatic hyperuricaemia into gout. Ann Rheumat Dis.

[CR19] Kopecna M, Blaschke H, Kopecny D, Vigouroux A, Koncitikova R, Novak O (2013). Structure and function of nucleoside hydrolases from Physcomitrella patens and maize catalyzing the hydrolysis of purine, pyrimidine, and cytokinin ribosides. Plant Physiol.

[CR20] Krumbeck JA, Rasmussen HE, Hutkins RW, Clarke J, Shawron K, Keshavarzian A (2018). Probiotic Bifidobacterium strains and galactooligosaccharides improve intestinal barrier function in obese adults but show no synergism when used together as synbiotics. Microbiome.

[CR21] Le Chatelier E, Nielsen T, Qin J, Prifti E, Hildebrand F, Falony G (2013). Richness of human gut microbiome correlates with metabolic markers. Nature.

[CR22] Li C, Hsieh MC, Chang SJ (2013). Metabolic syndrome, diabetes, and hyperuricemia. Curr Opin Rheumatol.

[CR23] Lim MY, Rho M, Song Y-M, Lee K, Sung J, Ko G (2014). Stability of gut enterotypes in Korean monozygotic twins and their association with biomarkers and diet. Sci Rep.

[CR24] Liu X, Lv Q, Ren H, Gao L, Zhao P, Yang X (2020). The altered gut microbiota of high- purine-induced hyperuricemia rats and its correlation with hyperuricemia. PeerJ.

[CR25] Love MI, Huber W, Anders S (2014). Moderated estimation of fold change and dispersion for RNA-seq data with DESeq2. Genome Biol.

[CR26] Maiuolo J, Oppedisano F, Gratteri S, Muscoli C, Mollace V (2016). Regulation of uric acid metabolism and excretion. Int J Cardiol..

[CR27] McMurdie PJ, Holmes S (2013). phyloseq: an R package for reproducible interactive analysis and graphics of microbiome census data. PLoS ONE.

[CR28] Mora D, Arioli S (2014). Microbial urease in health and disease. PLoS Pathog..

[CR29] Narang RK, Topless R, Cadzow M, Gamble G, Stamp LK, Merriman TR (2019). Interactions between serum urate-associated genetic variants and sex on gout risk: analysis of the UK Biobank. Arthritis Res Ther..

[CR30] Neogi T, Jansen TLTA, Dalbeth N, Fransen J, Schumacher HR, Berendsen D (2015). 2015 Gout classification criteria: an American College of Rheumatology/European League Against Rheumatism collaborative initiative. Ann Rheum Dis.

[CR31] Ogawa J (2006). Analysis of microbial purine metabolism and its application for hyperuricemia prevention. Div Appl Life..

[CR32] Parks D, Beiko R. STAMP: statistical analysis of metagenomic profiles. 2013 SRC:1–6.

[CR33] Quast C, Pruesse E, Yilmaz P, Gerken J, Schweer T, Yarza P (2012). The SILVA ribosomal RNA gene database project: improved data processing and web-based tools. Nucleic Acids Res..

[CR34] Ramazzina I, Folli C, Secchi A, Berni R, Percudani R (2006). Completing the uric acid degradation pathway through phylogenetic comparison of whole genomes. Nat Chem Biol..

[CR35] Ramazzina I, Costa R, Cendron L, Berni R, Peracchi A, Zanotti G (2010). An aminotransferase branch point connects purine catabolism to amino acid recycling. Nat Chem Biol.

[CR36] Riviere A, Selak M, Lantin D, Leroy F, Vuyst L (2016). De bifidobacteria and butyrate-producing colon bacteria: importance and strategies for their stimulation in the human gut. Front Microbiol.

[CR37] Salway JG (2018). The Krebs uric acid cycle: a forgotten Krebs cycle. Trends Biochem Sci..

[CR38] Shao T, Shao L, Li H, Xie Z, He Z, Wen C (2017). Combined signature of the fecal microbiome and metabolome in patients with gout. Front Microbiol.

[CR39] Shin N-R, Whon TW, Bae J-W (2015). Proteobacteria: microbial signature of dysbiosis in gut microbiota. Trends Biotechnol..

[CR40] Sorensen LB, Levinson DJ (1975). Origin and extrarenal elimination of uric acid in man. Nephron.

[CR41] Stoll ML, Pierce MK, Watkins JA, Zhang M, Weiss PF, Weiss JE (2019). Akkermansia muciniphila is permissive to arthritis in the K/BxN mouse model of arthritis. Genes Immun.

[CR42] Switzer A, Burchell L, McQuail J, Wigneshweraraj S (2020). The adaptive response to long-term nitrogen starvation in *Escherichia coli* requires the breakdown of allantoin. J Bacteriol..

[CR43] Vázquez-Mellado J, Cruz J, Guzmán S, Casasola-Vargas J, Lino L, Burgos-Vargas R (2006). Severe tophaceous gout. Characterization of low socioeconomic level patients from México. Clin Exp Rheumatol..

[CR44] Wang C-H, Zhang C, Xing X-H (2016). Xanthine dehydrogenase: an old enzyme with new knowledge and prospects. Bioengineered.

[CR45] Xi H, Schneider BL, Reitzer L (2000). Purine catabolism in *Escherichia coli* and function of xanthine dehydrogenase in purine salvage. J Bacteriol..

[CR46] Xi Y, Yan J, Li M, Ying S, Shi Z (2019). Gut microbiota dysbiosis increases the risk of visceral gout in goslings through translocation of gut-derived lipopolysaccharide. Poult Sci.

[CR47] Xu X, Li C, Zhou P, Jiang T (2016). Uric acid transporters hiding in the intestine. Pharm Biol..

[CR48] Yamauchi T, Oi A, Kosakamoto H, Akuzawa-Tokita Y, Murakami T, Mori H (2020). Gut bacterial species distinctively impact host purine metabolites during aging in *Drosophila*. iScience.

[CR49] Yang X, Yuan Y, Zhan C-G, Liao F (2012). Uricases as therapeutic agents to treat refractory gout: current states and future directions. Drug Dev Res..

[CR50] Yin J, Wei Y, Liu D, Hu Y, Lu Q, Ang EL (2019). An extended bacterial reductive pyrimidine degradation pathway that enables nitrogen release from β-alanine. J Biol Chem..

[CR51] Yishai O, Bouzon M, Döring V, Bar-Even A (2018). In vivo assimilation of one-carbon via a synthetic reductive glycine pathway in *Escherichia coli*. ACS Synth Biol..

[CR52] Zhang J, Kobert K, Flouri T, Stamatakis A (2014). PEAR: a fast and accurate Illumina Paired-End reAd mergeR. Bioinformatics.

