# Supplementary material for: Taxonomic variations in the gut microbiome of gout patients with and without tophi might have a functional impact on urate metabolism
Source: Mol Med. 2021 May 24;27:50. doi: 10.1186/s10020-021-00311-5 (PMC8142508; doi:10.1186/s10020-021-00311-5)
Supplement: Supplementary file 1 — Additional file 1: Table S1. Descriptive statistics and results of anthropometric, biochemical variables in gout groups and healthy controls. Text in bold denotes statistical significance. Age, BMI, and total cholesterol are expressed in mean and standard deviation, while the other continuous variables are summarized by median and interquartile range. a. P-value obtained from ANOVA. b. P-value obtained from Kruskall-Wallis test. c. P-value obtained from two tail Student’s T test. d. P-value obtained from Mann-Whitney’s U test. *Significant p-value obtained from Tukey post-hoc test comparing against control group & significant p-value obtained after multiple comparison correction comparing against control group. [file 10020_2021_311_MOESM1_ESM.docx]

**TABLE S1**

|  | **Healthy control** | **Chronic gouty arthritis**  **without tophi** | **Tophaceous gout** | ***P-value*** |
| --- | --- | --- | --- | --- |
| N | 53 | 25 | 33 |  |
| Age (years) |  |  |  |  |
| $\bar{x}(SD$) | 45.40 (7.93) | 50.76 (9.42) | **51.33 (13.88)*** | **0.02^a^** |
| BMI ( Kg/m^2^) |  |  |  |  |
| $\bar{x}(SD$) | 26.09 (2.71) | **30.22 (3.42)*** | 28.93 (5.12) | **<0.01^a^** |
| Glucose (mg/dL) |  |  |  |  |
| p50 (IQR) | 89.82 (17.92) | **97 (13.54)^&^** | 92.52 (10.69) | **0.02^b^** |
| Total cholesterol (mg/dL) |  |  |  |  |
| $\bar{x}(SD$) | 151.74 (41.88) | 164.46 (30.07) | 169.36 (33.21) | 0.09^a^ |
| Triglycerides (mg/dL) |  |  |  |  |
| p50 (IQR) | 127.31 (51.44) | **181 (115.68)^&^** | **179.8 (110.34)^&^** | **<0.01^b^** |
| Uric acid (mg/dL) |  |  |  |  |
| p50 (IQR) | 5.67 (1.24) | 6 (2.05) | 6.6 (3.9) | 0.06^b^ |
| Time of gout diagnosis |  |  |  |  |
| p50 (IQR) | --- | 6.5 (10) | 8 (15) | 0.63^d^ |
| Male gender (%) |  |  |  |  |
| N (%) | 46 (86.79) | 24 (96.00) | 33 (100.00) | 0.061^e^ |

Text in bold denotes statistical significance. Age, BMI, total cholesterol are expressed in mean and standard deviation, while the other continuous variable are summarized by median and interquartile range (IQR).

a. P-value obtained from ANOVA.

b. P-value obtained from Kruskall-Wallis test.

c. P-value obtained from two tail Student´s T test.

d. P-value obtained from Mann-Whitney´s U test.

* Significant p-value obtained from Tukey post-hoc test comparing against control group.

& significant p-value obtained after multiple comparison correction comparing against control group
