# Supplementary figures and images for: Taxonomic variations in the gut microbiome of gout patients with and without tophi might have a functional impact on urate metabolism
Source: Mol Med. 2021 May 24;27:50. doi: 10.1186/s10020-021-00311-5 (PMC8142508; doi:10.1186/s10020-021-00311-5)

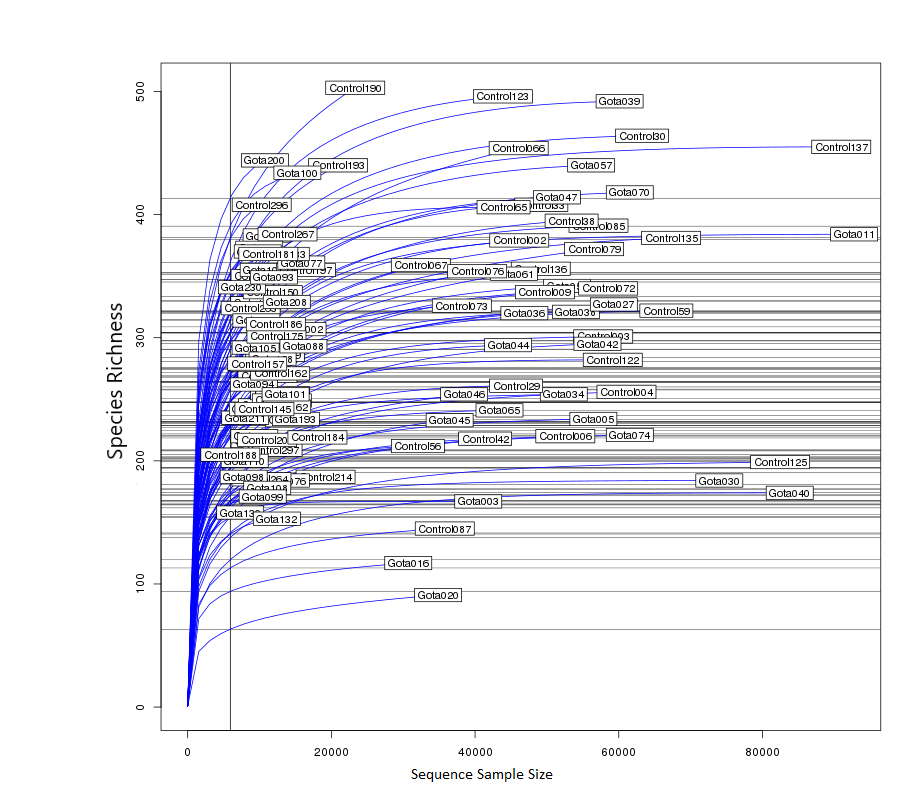

Supplement: Supplementary file 2 — Additional file 2: Fig. S1. Rarefaction analysis of the different samples. [file 10020_2021_311_MOESM2_ESM.png]

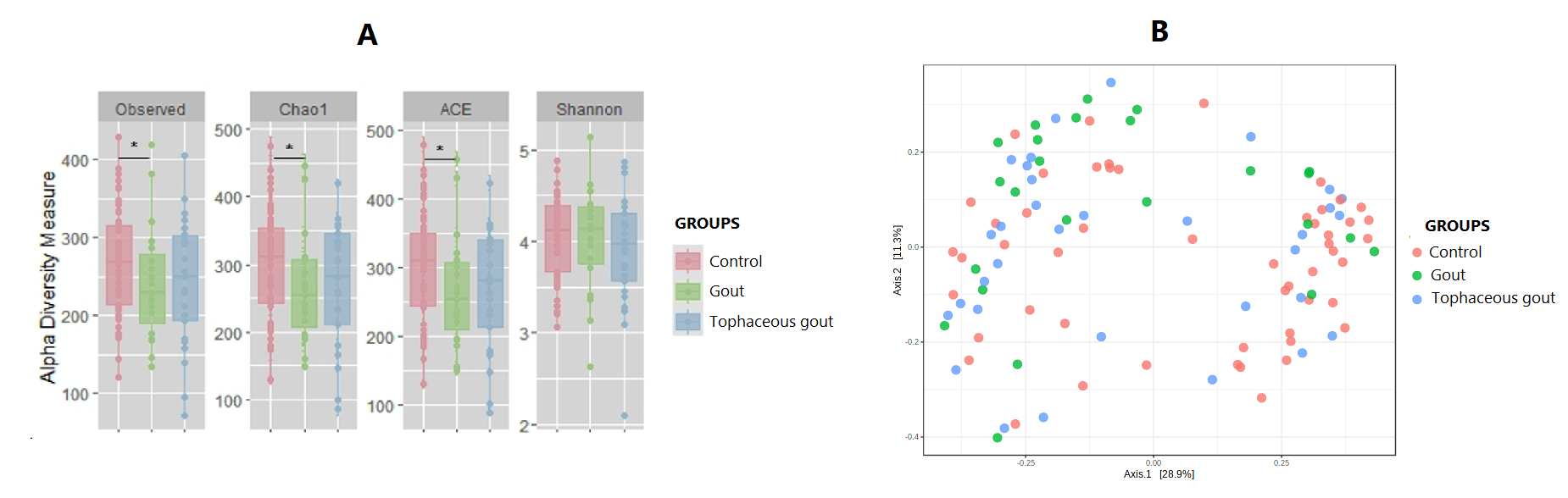

Supplement: Supplementary file 3 — Additional file 3: Fig. S2. A) Boxplot of Alpha-diversity indices without samples from patients with a BMI ≥ 30 kg/m2. S2B. B) Plot showed no clear clustering pattern between the gut microbiome of the study samples. [file 10020_2021_311_MOESM3_ESM.png]

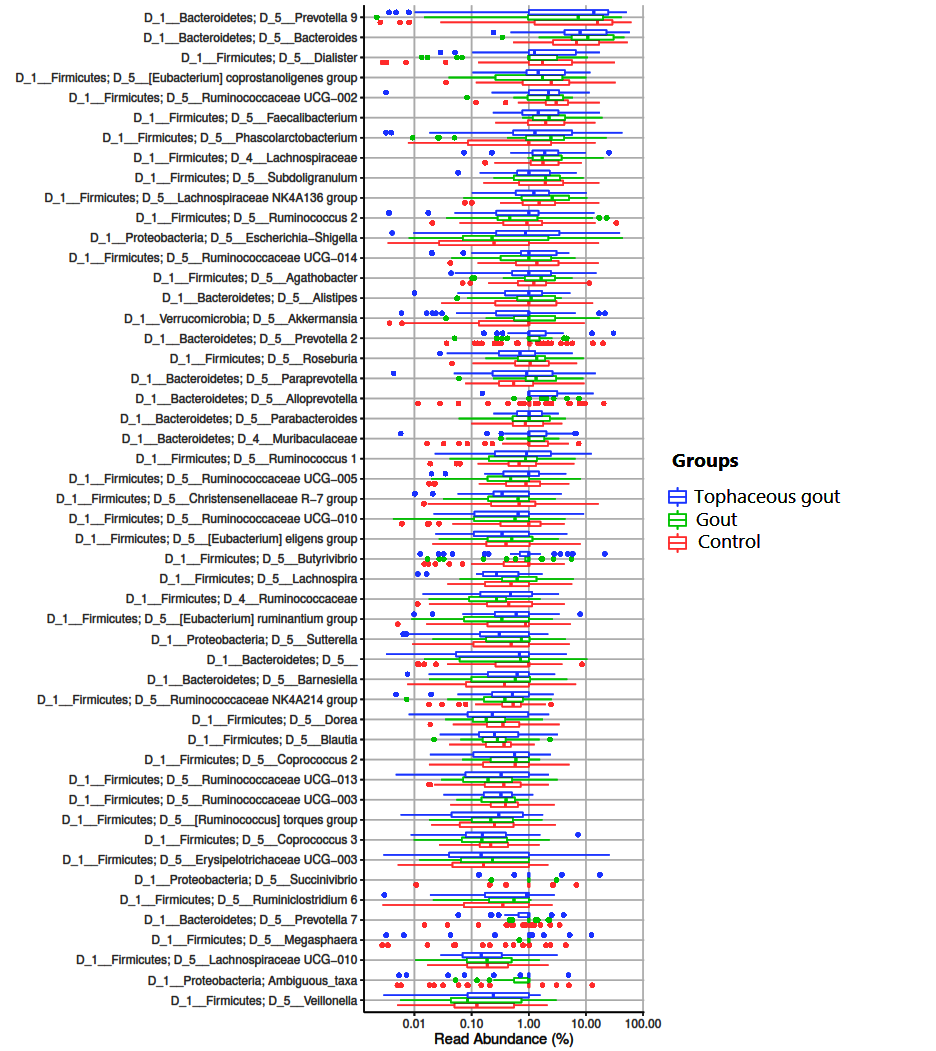

Supplement: Supplementary file 4 — Additional file 4: Fig. S3. Boxplot showing the occurrence of the top 50 most abundant genera in Mexican patients with gout and healthy controls. [file 10020_2021_311_MOESM4_ESM.png]

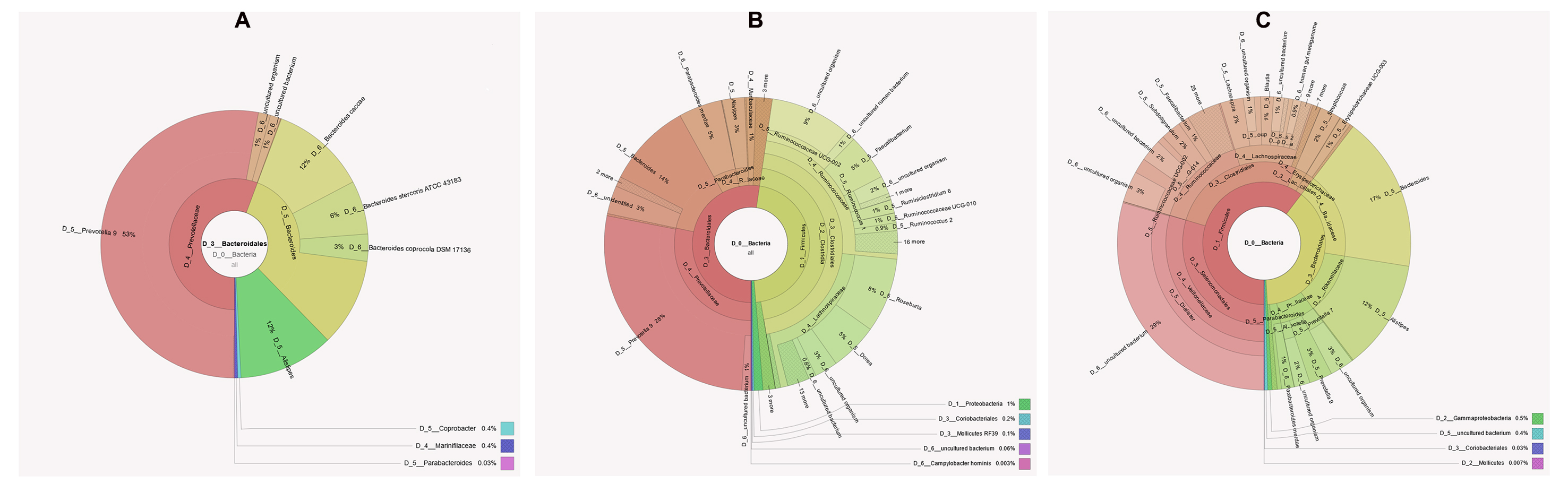

Supplement: Supplementary file 5 — Additional file 5: Fig. S4. Krona charts representing the taxonomic composition of the most abundant ASVs shared between the study groups. A) ASVs at the genus and species level shared between the two gout groups. B) ASVs shared between healthy controls and gout patients. C) ASVs shared between healthy controls and tophaceous gout patients. [file 10020_2021_311_MOESM5_ESM.jpg]
