# Supplementary material for: Taxonomic variations in the gut microbiome of gout patients with and without tophi might have a functional impact on urate metabolism
Source: Mol Med. 2021 May 24;27:50. doi: 10.1186/s10020-021-00311-5 (PMC8142508; doi:10.1186/s10020-021-00311-5)
Supplement: Supplementary file 6 — Additional file 6: Table S2. [file 10020_2021_311_MOESM6_ESM.docx]

**Table S2**

| **Bacterial Protein** | **KEGG enzyme ID** | **KEGG orthologue ID** | **Pathways** | **Enriched in** |
| --- | --- | --- | --- | --- |
| Urea carboxylase | EC:6.3.4.6 | K01941 | Arginine biosynthesis, atrazine degradation | HCs |
| Urease accessory protein | --- | --- | Nitrogen recycling |  |
| Urease subunit alpha | EC:3.5.1.5 | K01428 | Arginine biosynthesis, purine metabolism, atrazine degradation |  |
| Urease subunit gamma/beta | EC:3.5.1.5 | K14048 | Arginine biosynthesis, purine metabolism, atrazine degradation |  |
| Glycine dehydrogenase subunit 2 | EC:1.4.4.2 | K00281 | Amino-acids metabolism, glyoxylate and dicarboxylate metabolism  , biosynthesis of secondary metabolites, carbon metabolism | GPs |
| Glycine reductase complex component B subunit alpha and beta | EC:1.21.4.2 | K10671 | Glycine metabolism |  |
| Glycine reductase complex component B subunit gamma | EC:1.21.4.2 | K10672 | Glycine metabolism |  |
| Vitamin B12 transport system permease protein | --- | --- | Vitamin B12 transport | TGPs |
| Xanthine dehydrogenase YagR molybdenum-binding subunit | EC:1.17.1.4 | K00087 | Purine metabolism |  |
| Ribose 1,5-bisphosphokinase | EC:2.7.4.23 | K05774 | Pentose phosphate pathway |  |
| Xanthine dehydrogenase iron-sulfur-binding subunit | --- | K13480 | Purine metabolism |  |
| Vitamin B12 transport system substrate-binding protein | --- | --- | Vitamin B12 transport |  |
| Purine/pyrimidine-nucleoside phosphorylase | EC:2.4.2.1 2.4.2.2 | K09913 | Purine and Pyrimide metabolism, biosynthesis of secondary metabolites |  |
| Xanthine dehydrogenase FAD-binding subunit | EC:1.17.1.4 | K11177 | Purine metabolism |  |
| Nucleoside permease | --- | --- | Nucleoside Transport |  |
| 5-hydroxyisourate hydrolase | EC:3.5.2.17 | K07127 | Purine metabolism |  |
| (S)-ureidoglycine aminohydrolase | EC:3.5.3.26 | K14977 | Purine metabolism |  |
| Glycine cleavage system transcriptional repressor | --- | K03567 | Biofilm formation |  |
| Xanthine phosphoribosyltransferase | EC:2.4.2.22 | K00769 | Purine metabolism |  |
| Xanthosine phosphorylase | EC:2.4.2.- | K03815 | Purine metabolism |  |
| Methionine transaminase | EC:2.6.1.88 | K14287 | Amino-acids biosynthesis |  |
| Allantoate deiminase | EC:3.5.3.9 | K02083 | Purine metabolism |  |
| Purine nucleosidase | EC:3.2.2.1 | K01239 | Purine metabolism |  |

HCs: Healthy controls; GPs: gout patients; TGP: tophaceous gout patients
